# The association between healthy and unhealthy dietary indices with prostate cancer risk: a case-control study

**DOI:** 10.1186/s41043-024-00578-4

**Published:** 2024-06-20

**Authors:** Marzieh Mahmoodi, Baneen Chasib Gabal, Farzaneh Mohammadi, Fatma Magdi Ibrahim, Yahya Jalilpiran, Mehran Nouri, Shiva Faghih

**Affiliations:** 1 Student Research Committee, Shiraz University of Medical Sciences, Shiraz, Iran; 2 Nutrition Research Center, Department of Clinical Nutrition, School of Nutrition and Food Sciences, Shiraz University of Medical Sciences, Shiraz, Iran; 3 Medical Laboratory Technique College, the Islamic University, Najaf, Iraq; 4 Medical Laboratory Technique College, the Islamic University of Al Diwaniyah, Al Diwaniyah, Iraq; 5 Medical Laboratory Technique College, the Islamic University of Babylon, Babylon, Iraq; 6 Assistant professor, Community Health Nursing, RAK Medical and Health Sciences University, UAE .,; 7 Lecturer, geriatric nursing, Mansoura University, Mansoura, Egypt; 8 Department of Clinical Nutrition, School of Nutritional Science and Dietetics, Tehran University of Medical Sciences, Tehran, Iran; 9 Cancer Research Center, Health Research Institute, Babol University of Medical Sciences, Babol, Iran; 10 Health Policy Research Center, Institute of Health, Shiraz University of Medical Sciences, Shiraz, Iran; 11 Department of Community Nutrition, School of Nutrition and Food Sciences, Shiraz University of Medical Sciences, Shiraz, Iran

**Keywords:** Pro-healthy diet index, Non-healthy diet index, Pro-vegetarian dietary index, Prostate cancer, Iranian

## Abstract

**Introduction:**

According to our knowledge, the relationship between dietary patterns such as pro-healthy, pro-vegetarian, and non-healthy dietary patterns and prostate cancer risk has not been clearly investigated in Iranian men. Therefore, we aimed to investigate the relationship between adherence to a pro-healthy (PHDI), pro-vegetarian (PDP), and non-healthy dietary indices (NHDI) and the risk of prostate cancer.

**Method:**

In this matched case-control study, 125 participants (62 cases and 63 hospital-based controls) were enrolled from April to September 2015. Participants’ dietary intakes were evaluated using a valid and reliable 160-item semi-quantitative food frequency questionnaire. Dietary indices calculated based on previous studies. The relationship between dietary indices (PHDI, NHDI and PDP) and prostate cancer risk was assessed using binary regression models.

**Results:**

According to adjusted model, significant negative correlations were found between PHDI and PDP with prostate cancer (PHDI: OR = 0.31; 95% CI; 0.11–0.85; *P* = 0.023 – PDP: OR = 0.34; 95% CI; 0.15–0.75; *P* = 0.008). Also, a positive association was seen between NHDI and prostate cancer (OR = 3.01; 95% CI; 1.20–7.57; *P* = 0.019).

**Conclusion:**

We found that adherence to healthy dietary indices which includes high amounts of fruits, vegetables, and whole grains reduces the risk of prostate cancer. While adherence to a dietary pattern high in red and processed meat, refined grains, and sweetened beverages increases the risk of prostate cancer.

## Introduction

Prostate cancer has been increasing worldwide in recent years, especially in Asian countries [1]. Several factors affect the incidence of the disease, the most important of which are age, race, also genetic and environmental factors [2]. According to the epidemiological findings, environmental factors such as lifestyle, especially dietary habits, have an important effect on the risk of prostate cancer [3, 4]. In this regard, it has been reported that the risk of prostate cancer was higher in Japanese men who predominantly follow a Western dietary pattern [3]. Similarly, other findings have shown that Western dietary pattern has a positive association with the risk of prostate cancer [5]. In contrast, adherence to a healthy Mediterranean diet has protective effect against the risk of prostate cancer [5].

Loeb et al. showed that adherence to a plant-based diet with high intake of fruits, vegetables, nuts, legumes, and whole grains and low intake of animal products was associated with reduction in the risk of chronic diseases and mortality [6]. Evidence focused on the association between adherence to a plant-based diet and prostate cancer [7]. Epidemiological findings support the beneficial role of plant-based foods, especially tomatoes as the main sours of lycopene in reducing the risk of prostate cancer [6, 8]. Also, following the consumption of dairy products and red meat an increase in the risk of prostate cancer has been reported [6, 9, 10].

According to our knowledge, the relationship between pro-healthy, pro-vegetarian, and non-healthy dietary indices and prostate cancer risk has not clearly investigated in Iranian men. Therefore, the aim of the present study is to investigate the relationship between adherence to pro-healthy, pro-vegetarian, and non-healthy dietary indices and the risk of prostate cancer in Shiraz.

## Methods and materials

### Study design

The present case-control study was conducted from April to September 2015 in the urological disorders referral centers of two principal hospitals in Shiraz, Iran. The study protocol was reviewed and approved by the Ethics Committee of Shiraz University of Medical Sciences, Shiraz, Iran. This study was performed based on the Declaration of Helsinki guideline. All the participants were informed about the study, then consent forms were obtained. Details of the present study were published previously [2, 11, 12].

### Participants

One hundred and twenty five participants (62 cases and 63 hospital-based controls) were enrolled in this study from April to September 2015. Five participants (2 cases and 3 controls) were removed from the final analysis due to their poor response to the food frequency questionnaire (FFQ). The patients’ medical records were obtained from the hospitals’ cancer registry database, the most well-known and most-referred medical centers of southern provinces in Iran for all types of diseases, as well as cancer [13, 14]. Demographic information and dietary intakes of the participants were obtained through face-to-face interviews. Moreover, anthropometric indices (height and weight) were measured using standard protocols.

Inclusion criteria for the cases was being newly diagnosed with prostate cancer by a pathologist and being eligible for open or radical prostatectomy. The control group was selected from conditions other than cancer and diabetes, including the eye (*n* = 21), ear, nose, and throat (ENT) (*n* = 20), gastrointestinal (*n* = 9), kidney (*n* = 8), and neurological (*n* = 5) diseases simultaneously with the cases and from the same hospitals. Both case and control groups had no history of receiving a diet for cancer or other chronic diseases. Exclusion criteria were having total energy intake less than 800 or more than 4200 kcal/d or answering less than 70 items out of 160 items of the FFQ [15]. Cases and controls were matched in terms of age (5-year groups) and body mass index (BMI) (< 19, 19-24.99, 25-29.99, and ≥ 30 kg/m^2^).

### Demographic assessments

Demographic and lifestyle information were assessed through a questionnaire included information on age, ethnicity (Fars/non-Fars), education (illiterate and primary/diploma and academic), job (employment/unemployment), smoking status (smoker/non-smoker), medication use such as aspirin, antihyperlipemic and antihypertensive drugs (yes/no), and physical activity (low or never/moderate/high).

### Anthropometric assessments

Weight was measured without shoes in light clothing by a digital scale (Glamor BS-801, Hitachi, China) to the nearest 100gr. Height was also measured in a standing position, without shoes, while the heels, buttocks, and head were attached to the wall, by an inflexible tape measure to the nearest 0.1 cm. All measurement were done after diagnosis of cancer.

### Dietary intake assessment

Participants’ dietary intakes were evaluated using a valid and reliable 160-item semi-quantitative FFQ [16]. According to this questionnaire, the frequency of food consumption of the participants were recorded based on the portion sizes of the usual Iranian food as follows: “never or less than once a month”, “1 to 3 times a month”, “once a week”, “2 to 4 times a week”, “5 to 6 times a week”, “once a day”, “2 to 3 times a day”, “4 to 5 times a day”, and “6 times or more a day”. Also, the portion sizes were categorized as small (half the stated average consumption or less), medium (equal to the stated average consumption), and large (one and a half times the stated average consumption or more). Afterward, the FFQs were analyzed by Borland Delphi Professional, version 7.0 and Visual Basic 2008 (VB 9.0) software. Furthermore, foods were converted into nutrients by Nutritionist 4 software (First Databank Inc. San Bruno, CA, USA). The frequency of food consumption was categorized on 6 scale with the following values: 1 (never), 2 (1 to 3 times a month), 3 (once a week), 4 (several times a week), 5 (once a day) and 6 (several times a day). The original ranks were then changed to real numbers stating the daily frequency of food consumption (as times/day) using the following method: never (0), 1 to 3 times a month (0.06), once a week (0.14), several times a week (0.5), once a day (1) and several times a day (2) [17, 18].

### Pro-healthy Diet Index assessment

The pro-healthy diet index (PHDI-10) measures the daily frequency consumption of 10 food groups which have beneficial health effects, including wholemeal bread, oatmeal, barley, whole grain pasta, thickly-ground barley, milk, fermented dairy products, fresh cheese, white meat, fish, seeds, legumes, fruits, and vegetables. To calculate PHDI-10 score, food groups intakes were divided into tertiles and scores 0–2 were assigned to first to last tertiles. The total score of PHDI-10 ranged 0–20. Higher scores means greater positive impact on health [17, 18].

#### Non-healthy Diet Index assessment

The non-healthy diet index (NHDI-14) values 0–28 to evaluate how often an individual consumes 14 different food groups per day. These food products are white bread, refined cereal products (such as plain pasta, white rice, and finely-ground barley), fried food, fast food, butter, hard and processed cheeses, processed meat products (such as cold cuts, sausages, and hot dogs), red meat dishes, canned meat, sweets, confectionery, sweetened beverages (carbonated or non-carbonated), and alcoholic beverages. It is worth noting that in the current study, consumption of lard, alcoholic beverages, and energy drinks was not taken into account in the calculation of the index due to religious considerations in Iranian society. NHDI-14 food groups converted to tertiles and then score 0 was considered for the first, score 1 for the second and score 2 for last tertile. The total score of NHDI ranged 0–22 based of Iranian FFQ items. The higher the score, the greater the negative impact on the health [17, 18].

#### Provegetarian Dietary Pattern (PDP) assessment

The PDP comprises a range of 12–60 and consists of 12 different food groups. These include 7 vegetable groups and 5 animal-based food groups. The vegetable groups consist of fruits, vegetables, cereals, legumes, potatoes, nuts, and olive oil, and the animal-based food groups consist of meat/meat products, animal fat for cooking or as a spread, fish and other seafood, dairy products, and eggs. To score the consumption of vegetable groups in grams per day, they were divided into quintiles numbered 1–5. The sum of these quintiles ranged from 7 to 35. The consumption of animal-based food groups in grams per day was scored in reverse, with quintiles numbered 1–5 in reverse order. The total of these quintiles ranged from 5 to 25 [19].

### Statistical analysis

Data analysis was performed using SPSS version 23 software (SPSS Inc. Chicago, IL, USA). A P-value less than 0.05 was considered significant. The dietary indices were categorized into two groups, including below and equal to the median (reference) and above the median and reported as the median (interquartile range (IQR). Other variables were reported as mean ± SD and percentage. Mann-Whitney and Independent sample t-tests were used to compare continuous variables, and the Chi-square test was used to compare categorical variables between two groups. The relationship between dietary indices (PHDI, NHDI and PDP) and prostate cancer risk was assessed using logistic regression. Education, smoking status, physical activity and energy intake were considered as potential confounders and the results were reported as odd ratios (OR) and 95% confidence intervals (CI).

## Results

Demographic, anthropometric, and dietary characteristics of the participants are shown in Table 1. Significant differences were observed between NHDI score (*P* = 0.016), PDP score (*P* = 0.002) and physical activity (*P* = 0.024) of the case and control groups.

**Table 1:** The characteristic of the study participants

Variables	Cases (60)	Controls (60)	*P*-value
Age (year) ^^^			0.210
˂ 60 year	7 (11.7)	8 (13.3)	
60–65 year	18 (30.0)	27 (45.0)	
65–70 year	24 (40.0)	14 (23.3)	
≥ 70 year	11 (18.3)	11 (18.4)	
BMI (kg/m^2^) ^*^	24.8 ± 3.6	25.8 ± 3.4	0.121
Energy (kcal/day) ^*^	2712.2 ± 593.5	2596.1 ± 712.7	0.334
Fiber (g/day) ^^^	20.1 (8.8)	23.3 (11.6)	0.187
PHDI total score ^^^	10.0 (4.0)	11.0 (7.0)	0.552
NHDI total score ^^^	12.0 (4.0)	10.0 (5.0)	**0.016**
PDP total score	34.0 (6.7)	37.0 (6.0)	**0.002**
Ethnicity, % ^&^			0.825
Fars	80.0	76.7	
Not Fars	20.0	23.3	
Job, % ^&^			0.711
Employment	56.7	61.7	
Unemployment	43.3	38.3	
Education, % ^&^			0.134
Illiterate and Primary	68.3	53.3	
Diploma and Academic	31.7	46.7	
Smoking, % ^&^			0.833
No	76.7	73.3	
Yes	23.3	26.7	
Physical activity, % ^&^			**0.024**
Never or Less	38.3	20.0	
Moderate	41.7	40.0	
High	20.0	40.0	
Lipid medication, % ^&^			1.000
No	90.0	90.0	
Yes	10.0	10.0	
HTN medication, % ^&^			0.302
No	68.3	78.3	
Yes	31.7	21.7	
Aspirin use, % ^&^			0.369
No	83.3	75.0	
Yes	16.7	25.0	

BMI: body mass index, PHDI: pro-healthy diet index, NHDI, non-healthy diet index, PDP: provegetarian dietary pattern

^^^Using Mann-Whitney for abnormal continuous variables and values are reported median (IQR)

^*^Using independent samples T-test for normal continuous variables and values are reported mean ± SD

^&^Using chi-square test for categorical and values are reported percent

As presented in Tables 2 and 3, the intakes of fruits (*P* = 0.003) and vegetables (*P* = 0.009) food groups were higher, but red meats (P ˂ 0.001) and sweets and confectionary (*P* = 0.002) intakes were lower in the control group.

**Table 2:** Pro-healthy components intake by case and control group

Food Components	Cases (60)	Controls (60)	*P*-value
	Median (IQR)	Median (IQR)	
Whole Meal Breads (gr/day)	18.9 (116.0)	18.9 (78.9)	0.733
Other Whole Grain Cereal Products (gr/day)	0.0 (5.0)	0.0 (5.0)	0.317
Milk (gr/day)	98.1 (131.7)	97.6 (124.0)	0.391
Fermented Dairy Products (gr/day)	232.5 (169.3)	215.0 (216.4)	0.799
Fresh Cheese (gr/day)	21.7 (9.0)	18.0 (27.0)	0.963
White Meat (gr/day)	55.0 (35.0)	54.0 (24.0)	0.461
Fish (gr/day)	38.5 (54.0)	19.2 (48.1)	0.283
Legume (gr/day)	50.0 (25.0)	42.0 (30.0)	0.295
Fruit (gr/day)	275.1 (196.7)	406.7 (353.7)	**0.003**
Vegetables (gr/day)	580.1 (229.7)	667.2 (404.2)	**0.009**

Using Mann-Whitney test

Values are reported median (IQR)

**Table 3:** Non-healthy components intake by case and control group

Food Components	Cases (60)	Controls (60)	*P*-value
	Median (IQR)	Median (IQR)	
White Bread (gr/day)	75.0 (109.2)	41.5 (103.6)	0.960
Other Purified Cereal Products (gr/day)	294.6 (103.3)	294.9 (107.0)	0.548
Fast Food (gr/day)	0.0 (0.0)	0.0 (0.0)	0.403
Fried Foods (gr/day)	11.0 (28.7)	11.0 (22.0)	0.760
Butter (gr/day)	1.0 (3.0)	0.0 (2.0)	0.405
Hard and Processed Cheeses (gr/day)	0.0 (14.1)	0.0 (10.5)	0.088
Processed Meat Products (gr/day)	0.0 (0.0)	0.0 (0.0)	0.928
Red Meat (gr/day)	72.0 (42.1)	43.5 (38.5)	**˂0.001**
Sweets and Confectionary (gr/day)	27.2 (25.0)	16.7 (25.2)	**0.002**
Canned Meat (gr/day)	0.0 (6.2)	6.1 (12.3)	0.105
Sweetened Carbonated or Non-carbonated Beverages (gr/day)	30.9 (70.3)	32.5 (44.9)	0.372

Using Mann-Whitney test

Values are reported median (IQR)

According to crude model, a significant relationship was observed between prostate cancer risk and NHDI (OR = 2.77; 95% CI (1.32–5.85); *P* = 0.007) also an inverse relation with PDP (OR = 0.34; 95% CI (0.16–0.72); *P* = 0.005). After adjusting for potential confounding factors, significant negative correlations were found between PHDI (OR = 0.31; 95% CI; 0.11–0.85; *P* = 0.023) and PDP (OR = 0.34; 95% CI; 0.15–0.75; *P* = 0.008) with prostate cancer risk. Also, a positive association was seen between NHDI and prostate cancer risk (OR = 3.01; 95% CI; 1.20–7.57; *P* = 0.019) (Table 4).

**Table 4:** Associations between pro- and non-healthy diet index and pro-vegetarian diet pattern with prostate cancer

Median of indices	Case/Control	Crude Model			Adjusted Model	
		OR		95% CI	OR	95% CI
**Pro-healthy diet index**						
M_1_ (≤ 10)	38/29	1.00		Ref.	1.00	Ref.
M_2_ (˃ 10)	22/31	0.56		0.29–1.16	**0.31**	**0.11–0.85**
P-value		0.120			**0.023**	
**Non-healthy diet index**						
M_1_ (≤ 11)	22/37	1.00		Ref.	1.00	Ref.
M_2_ (˃ 11)	38/23	**2.77**		**1.32–5.85**	**3.01**	**1.20–7.57**
P-value		**0.007**			**0.019**	
**Provegetarian Dietary Pattern**						
M_1_ (≤ 35)	39/23	1.00	Ref.		1.00	Ref.
M_2_ (˃ 35)	21/37	**0.34**	**0.16–0.72**		**0.34**	**0.15–0.75**
P-value		**0.005**			**0.008**	

Model 1: crude model

Model 2: adjusted for education, smoking, physical activity and energy intake

Obtained from logistic regression

These values are odd ratio (95% CIs)

Significant values are shown in bold

## Discussion

In the current study, two identified dietary indices including the PHDI, and PDP were associated with reduced risk of prostate cancer, while the NHDI was potentially associated with an increase in the risk of prostate cancer.

Pro-vegetarian and pro-healthy dietary indices are rich in fruits, vegetables, legumes, fish, fermented dairy products, and whole grains. In contrast, the non-healthy diet index is rich in refined grains, red and processed meat products, fast foods, fried foods, butter, sweets, and sweetened carbonated or non-carbonated beverages. Recent evidence has shown that a healthy dietary pattern as a nutrient dense diet is associated with a reduction in the risk of prostate cancer but an unhealthy dietary pattern as an energy dense diet is reversely associated [5].

These findings are consistent with the results of several studies. In a case-control study conducted by Askari et al. on men in Tehran, Iran, a significant increase in the risk of prostate cancer following adherence to a Western diet and a significant decrease following adherence to a healthy diet was observed [20]. In another case-control study in Australia by Ambrosini et al., adherence to the western dietary pattern was associated to the risk of prostate cancer [21]. Moreover, in the case-control study which was conducted in Kermanshah, Bagheri et al. reported that after adjusting for confounders such as alcohol consumption, smoking status, level of total energy intake, and physical activity and education, adherence to a healthy dietary pattern was associated with a significant reduction in the risk of prostate cancerbut adherence to an unhealthy dietary pattern was related to higher risk [5]. Similarly, two other studies also reported that adherence to unhealthy dietary patterns such as western diet was attributed to the increased risk of prostate cancer risk [22, 23].

In this view, it has been shown that greater adherence to the Western dietary pattern, as a diet rich in red and processed meat, saturated fats, and refined grains could increase the risk of prostate cancer [2]. This effect is attributed to the high content of energies and fats, heterocyclic amines, and oxidizing agents of unhealthy diets [5]. In addition, these food items, including carbonated drinks and sweet beverages, have high glycemic index, which increases the risk of cancer by causing hyperinsulinemia [24]. Some findings have also approved that due to their high energy content, unhealthy diets could increase the production of growth factors and angiogenesis which increased the risk of prostate cancer [24].

In contrast, the protective effects of healthy dietary patterns such as Healthy eating index (HEI) against prostate cancer has been reported [23]. This association may be explained by several mechanisms. High intakes of fruits and vegetables in healthy dietary patterns have chemo-preventive effects through their antioxidants compounds [25]. Also, bioactive compounds content of the plant-based diets such as fiber, phenol, polyphenol, and other antioxidant vitamins have anti-cancer effects [26].

As oxidative stress plays a key role in prostate cancer pathogenesis [27], antioxidant compounds have beneficial effects on its risk by reducing the oxidative stress status [28] [27]. Moreover, the consumption of fruits and vegetables as rich sources of lutein plays important role in reducing the risk of prostate cancer due to the antioxidant effects, regulation of apoptosis, and angiogenesis [28]. Receiving high amount of vitamin K through vegetables also has anti-cancer effects via reducing oxidative stress and activating the apoptosis pathway [28]. In this regard, it has been reported that antioxidant and phenolic compounds, have anti-inflammatory as well as protective effects against DNA damage-induced oxidative conditions [29].

In general, the intake of dietary fiber has a protective effect against cancer by slowing down the transit time and increasing the binding of carcinogens, also the production of short-chain fatty acids [29, 30]. Besides, high intake of dietary fiber in healthy plant-based diets could reduce the risk of prostate cancer by improving insulin sensitivity and reducing the bioavailability of insulin-like growth factor-1 (IGF-1) [28]. Similar results have been reported in other cohort and case-control studies for the protective effects of dietary fiber intake against prostate cancer [31, 32].

The present study has several strengths, first of all effect of pro-healthy, pro-vegetarian, and non-healthy dietary indices on the risk of prostate cancer in Iranian men was done for the first time. Valid and reliable questionnaire have also been used to collect data related to dietary intakes. Furthermore, multiple logistic regression models used to adjust several confounders. However, there are several weaknesses in this study that should be note. The small sample size, along with the use of the FFQ questionnaire, which is a retrospective tool and may lead to risk of recall bias, are important weaknesses of the study. Moreover, the FFQ is a tool to measure participants’ usual food consumption in the past year, which seems to be a limitation for discussing the long-term effects of diet on prostate cancer risk.

In conclusion, we found that adherence to healthy dietary indices which include high amounts of fruits, vegetables, and whole grains reduces the risk of prostate cancer. While adherence to a diet high in red and processed meat, refined grains, and sweetened beverages increases the risk of it.

## Data Availability

No datasets were generated or analysed during the current study.
